# Supplementary material for: Calibration of PurpleAir low-cost particulate matter sensors: model development for air quality under high relative humidity conditions
Source: Atmos Meas Tech. Author manuscript; Available in PMC 2025 Mar 12. (PMC11900072; doi:10.5194/amt-17-6735-2024)
Supplement: Mathieu-Campbell et al 2024 suppl [file NIHMS2049770-supplement-Mathieu-Campbell_et_al_2024_suppl.pdf]

Supplement of Atmos. Meas. Tech., 17, 6735–6749, 2024
https://doi.org/10.5194/amt-17-6735-2024-supplement
© Author(s) 2024. CC BY 4.0 License.

Atmospheric Measurement Techniques
Open Access

Supplement of

Calibration of PurpleAir low-cost particulate matter sensors: model development for air quality under high relative humidity conditions

Martine E. Mathieu-Campbell et al.

Correspondence to: Jennifer Richmond-Bryant (jrbryan3@ncsu.edu)

The copyright of individual parts of the supplement might differ from the article licence.

SUPPLEMENTAL MATERIAL

List of tables and figures

Table S1: Percentage of hourly data removed by QA process from the initial 56 PurpleAir sensors
Table S2: MLR model development (model fit using hourly data) for the 1.0-km radius
Table S3: SSC model development for the 1.0-km radius
Table S4: MLR model development (model fit using hourly data) for the 2.0-km radius
Table S5: SSC model development for the 2.0-km radius
Table S6: LGOCV results – MLR models
Table S7: LGOCV results – SSC models
Table S8: LOSOCV results using Model 4 from MLR and SSC
Table S9: Methods evaluated to determine the optimal number of clusters using NbClust
Table S10: MLR and SSC model development (model fit using hourly data) for the 0.5-km radius
Table S11: Evaluation of the performance of the models by AQI category
Table S12: Summary table of the evaluation of the AQI per model for the daily dataset
Table S13: List of the PurpleAir sensors and AQS used in the study with the estimated distance between stations
Table S14: Tests of model coefficient precision

Figure S1: Correlation graphs between RH from each PA sensor and RH from the nearest NOAA sensor (Table S13).
Figure S2: Correlation graphs between RH from PA sensors and RH from the nearest NOAA sensor to each PA sensor. a) All the 21 PA sensors for the 0.5-km radius, b) All PA sensors except r), s) and t) from Figure S1.
Figure S3: Distribution of RH and T from PurpleAir data for our three buffers (0.5 km, 1.0 km and 2.0 km)
Figure S4: Distribution of RH and T from PurpleAir data for our three buffers (0.5 km, 1.0 km and 2.0 km).
Figure S5: Correlation between DP from PurpleAir and NOAA

1 Data cleaning process

Table S1: Percentage of hourly data removed by QA process from the initial 56 PurpleAir sensors

QA criteria	% removed*
Process 1: Removing NAs (PM, T, RH)	2.026
Process 2: Channels A & B agreement	
Low concentration (≤ 25 µg m^{-3}): 537,246 obs.	2.242
High concentration (>25 µg m^{-3}): 80,196 obs.	2.056
Process 3: A & B concentration < 1.5 µg m^{-3}	6.753
Process 4: Average A & B concentration > 1000 µg m^{-3}	0.005
Process 5: Removing data from sensors with RH issues	5.527
Process 6: Removing RH≠ 0-100 % and T ≠ 0-130 °F	3.484

* percent removed from the total number of observations

The data cleaning process 5 allowed to remove faulty PA sensors (Figures S1 and S2). The 21 graphs correspond to the 21 PA sensors for the 0.5-km radius. R, n and m correspond to the estimated Pearson correlation, the number of data points and the slope of the linear regression.

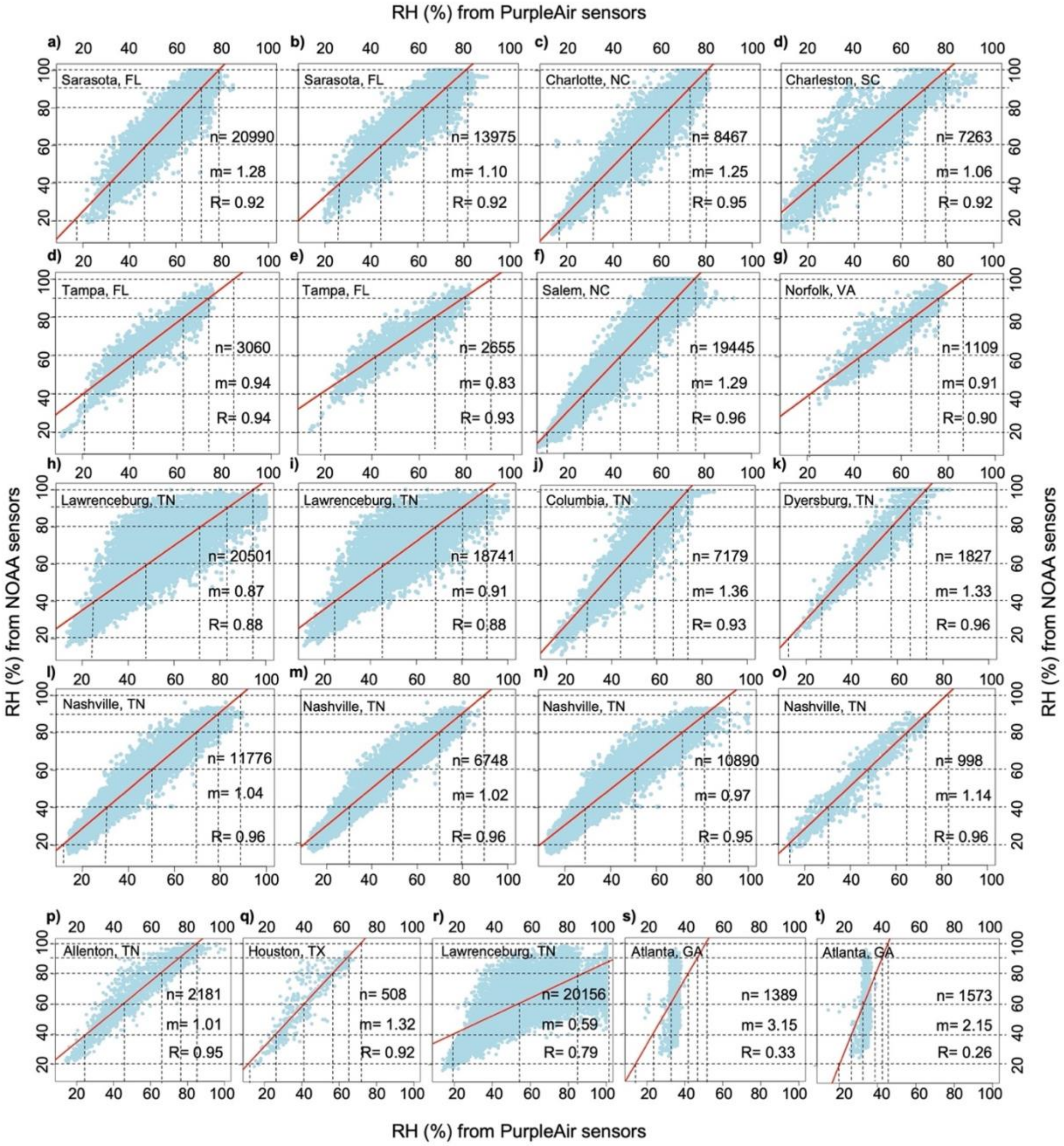

Figure S1: Correlation graphs between RH from each PA sensor and RH from the nearest NOAA sensor (Table S13).

Except for Figures S1-s and S1-t that present a very low Pearson correlation R, every individual PA displays a correlation R varying between 79 % and 96 % with 16/21 PA sensors presenting an R equal or greater than 90 %. As reported by recent studies (Tryner et al., 2020, Magi et al., 2020, Giordano et al., 2021; Barkjohn et al., 2022), PA sensors tend to report dryer humidity measurements than ambient conditions with a general difference of 10 % to 20 % for our sensors (Figure S2). However, Figure S1-r shows that one of our PA sensors reported more humid measurements for RH values of 80 % or greater.

Moreover, the slope of the linear regression estimated for each PA sensor (Figure S1) shows that RH from Figures S1-r, S1-s and S1-t exhibit the larger bias metrics. Figure S1-r shows that RH from the PA sensor tend to underestimate ambient RH while PA sensors represented in Figures S1-s and S1-t tend to more than doubled or tripled RH values from NOAA.

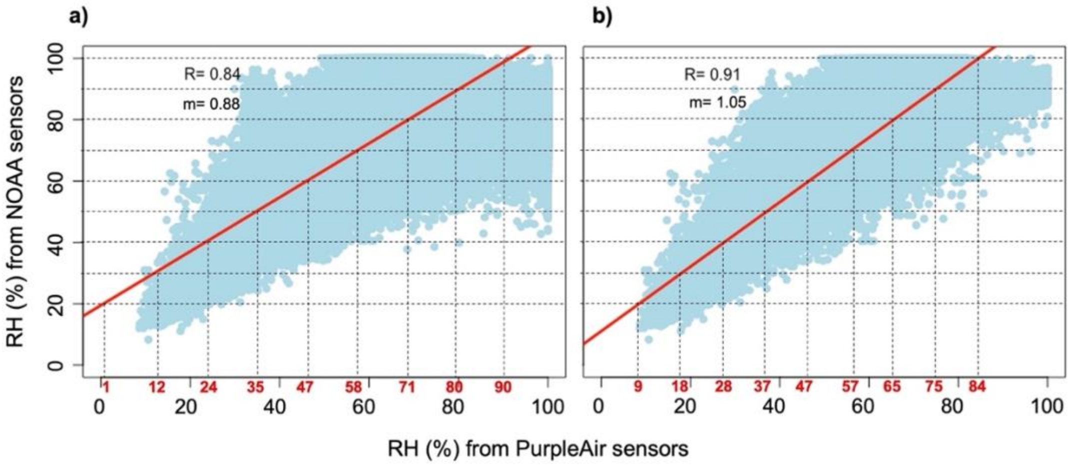

Figure S2: Correlation graphs between RH from PA sensors and RH from the nearest NOAA sensor to each PA sensor. a) All the 21 PA sensors for the 0.5-km radius, b) All PA sensors except r), s) and t) from Figure S1.

2 Evaluating our PurpleAir meteorological data

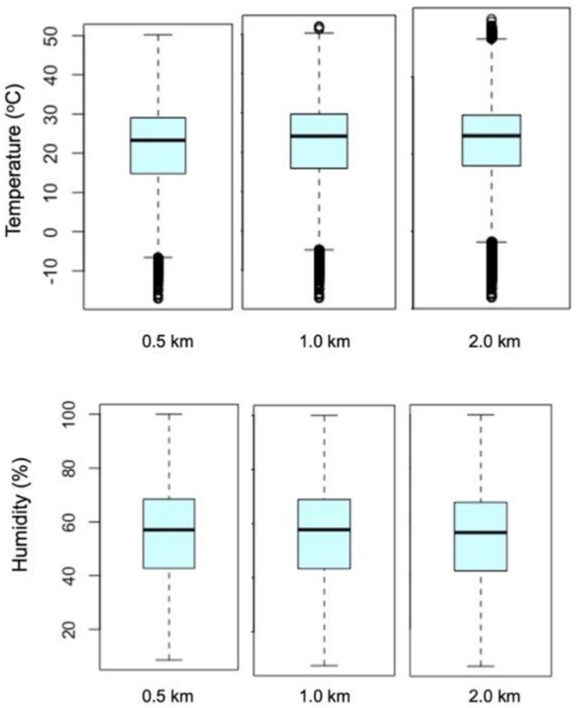

Figure S3: Distribution of RH and T from PurpleAir data for our three buffers (0.5 km, 1.0 km and 2.0 km)

Figure S4 shows that non-linearity in the curve started around RH of 50 %. PurpleAir datapoints that fell within a range of RH less or equal to 50 % are in green and those that fell within a range greater than 50 % are shown in blue.

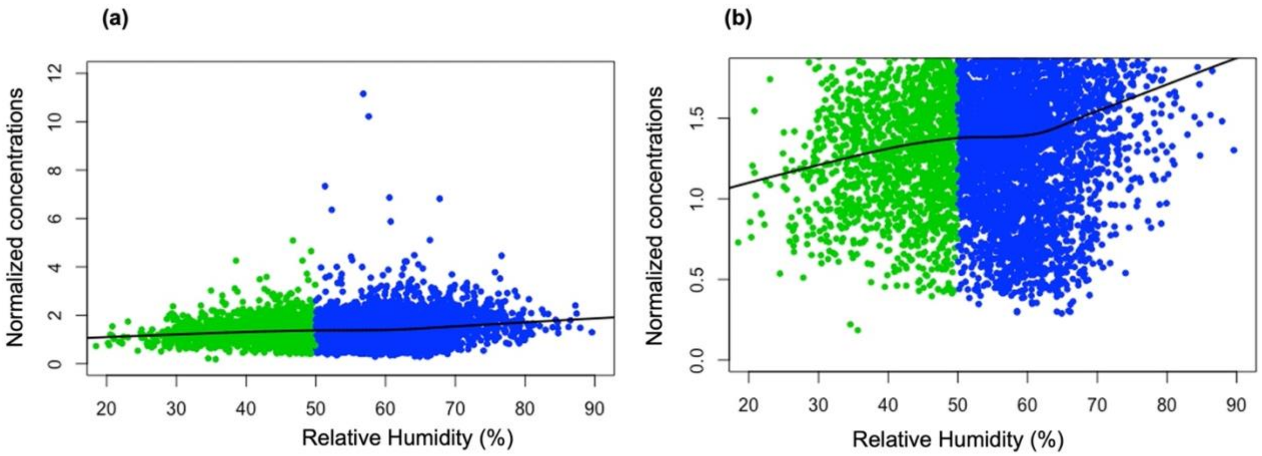

Figure S4: Correlation between the ratio of raw $PM_{2.5}$ PurpleAir and AQS concentrations and RH showing the nonlinearity of $PM_{2.5}$ PurpleAir concentrations. Graph a) represents the entire dataset, and graph b) is a zoom in to better display the regression line and the nonlinearity of the data.

3 Model fit using the 1.0-km radius dataset

Table S2: MLR model development (model fit using hourly data) for the 1.0-km radius

**Parameters**		**Model fit with hourly data**			
**Models**		**R^2 (%)**	**RMSE (μg m^{-3})**	**MAE (μg m^{-3})**	**R (%)**
**Model 1**	4.3410721+0.3796856 PA_i	58	3.85	2.47	76
**Model 2**	6.9941051+0.3872666 PA_i -0.0489237 RH_i	60	3.76	2.40	77
**Model 3**	1.6915454+0. 3849136 PA_i +0. 1149728 T_i	62	3.68	2.39	78
**Model 4**	4.1204142+0. 3907494 PA_i -0. 0405732 RH_i + 0. 1050501 T_i	63	3.61	2.32	79
**Model Bj**	5.72+0.524 PA_i -0.0852 RH_i	60	4.40	2.94	77

Table S3: SSC model development for the 1.0-km radius

Parameters		Model fit with hourly data			
Clusters (Number of observations)	Models	R^2 (%)	RMSE (μg m^{-3})	MAE (μg m^{-3})	R (%)
RH ≤ 50 (85616)	2.782329 + 0.368994 PA_i - 0.010616 RH_i + 0.122888 T_i	57	3.94	2.43	75
RH >50 (152431)	5.1538241 + 0.3980145 PA_i - 0.0539108 RH_i + 0.0943790 T_i	67	3.40	2.26	82

4 Model fit using the 2.0-km radius dataset

Table S4: MLR model development (model fit using hourly data) for the 2.0-km radius

Parameters		Model fit with hourly data			
Models		R^2 (%)	RMSE (μg m^{-3})	MAE (μg m^{-3})	R (%)
**Model 1**	4. 7265899 +0. 3763097 PA_i	55	4.30	2.72	74
**Model 2**	7. 5916138 +0. 3844184 PA_i -0. 0545752 RH_i	58	4.19	2.63	76
**Model 3**	1. 7548043 +0. 3803865 PA_i +0. 1250425 T_i	59	4.13	2.62	77
**Model 4**	4. 3418026 +0. 3862249 PA_i -0. 0425548 RH_i + 0. 1101893 T_i	60	4.06	2.55	78
**Model Bj**	5.72+0.524 PA_i -0.0852 RH_i	58	4.84	3.10	76

Table S5: SSC model development for the 2.0-km radius

Parameters		Model fit with hourly data			
Clusters (Number of observations)	Models	R^2 (%)	RMSE (μg m^{-3})	MAE (μg m^{-3})	R (%)

RH ≤ 50 (154276)	2.6452739 + 0.3676529 PA_i - 0.0057266 RH_i + 0.1303605 T_i	54	4.45	2.74	74
RH >50 (239734)	6.0381100 + 0.3926179 PA_i - 0.0646265 RH_i + 0.0961319 T_i	65	3.77	2.42	80

5 Cross-validation using LGOCV and LOSOCV for the 0.5-km radius

Table S6: LGOCV results – MLR models

**Parameters**		**Model fit with hourly data**			
**Models**		**R^2 (%)**	**RMSE ($\mu g\ m^{-3}$)**	**MAE ($\mu g\ m^{-3}$)**	**R (%)**
**Model 1**	3.6667550+0.4053418 PA_i	69	3.19	2.13	83
**Model 2**	6.3384228+0.4143437 PA_i -0.0506037 RH_i	71	3.06	2.05	84
**Model 3**	1.7642336+0.4109897 PA_i +0.0847196 T_i	71	3.05	2.06	84
**Model 4**	4.3295358+0.4182906 PA_i -0.0445768 RH_i + 0.0752867 T_i	73	2.95	1.98	85

Table S7: LGOCV results – SSC models

**Parameters**		**Model fit with hourly data**			
**Clusters (Number of observations)**	**Models**	**R^2 (%)**	**RMSE ($\mu g\ m^{-3}$)**	**MAE ($\mu g\ m^{-3}$)**	**R (%)**
RH ≤ 50 (59405)	2.738732 + 0.425834 PA_i - 0.008944 RH_i + 0.079210 T_i	71	2.93	1.86	84
RH >50 (100243)	7.230374 + 0.412683 PA_i - 0.085278 RH_i + 0.070655 T_i	74	2.92	2.02	86

Table S8: LOSOCV results using Model 4 from MLR and SSC

Data test State	MLR model		SSC model			
	Model 4		Cluster 1		Cluster 2	
	RMSE ($\mu g\ m^{-3}$)	MAE ($\mu g\ m^{-3}$)	RMSE ($\mu g\ m^{-3}$)	MAE ($\mu g\ m^{-3}$)	RMSE ($\mu g\ m^{-3}$)	MAE ($\mu g\ m^{-3}$)
**FL**	3.02	2.03	2.42	1.48	3.19	2.21
**NC**	2.86	1.89	2.89	1.78	2.71	1.85
**TN**	3.26	2.37	3.12	2.1	3.27	2.43
**SC**	3.43	1.92	4.03	1.85	2.95	1.9
**VA**	2.73	2.39	3.16	2.9	2.62	2.24
**TX**	4.6	3.16	5.1	3.68	3.79	2.37
**Average**	3.32	2.29	3.45	2.30	3.09	2.17

6 Estimating the optimal number of clusters

Among all indices:
Eight (8) proposed 2 as the best number of clusters.
Two (2) proposed 3 as the best number of clusters.
Two (2) proposed 4 as the best number of clusters.
Seven (7) proposed 5 as the best number of clusters.
Two (2) proposed 6 as the best number of clusters.
One (1) proposed 13 as the best number of clusters.
Two (2) proposed 14 as the best number of clusters.

Table S9: Methods evaluated to determine the optimal number of clusters using NbClust

#	Methods	Number of clusters	Value Index
1	KL	6	12.525
2	CH	5	2455.144
3	Hartigan	5	1479.653
4	CCC	2	86.151
5	Scott	5	2750.553
6	Marriot	5	349227082927.000
7	TrCovW	3	107827250739.000
8	TraceW	5	135580.700
9	Friedman	14	52.457
10	Rubin	5	11.169
11	Cindex	6	0.253
12	DB	2	0.882
13	Silhouette	2	0.384
14	Duda	2	0.723

15	PseudoT2	2	1043.876
16	Beale	2	0.381
17	Ratkowsky	4	0.379
18	Ball	3	195959.500
19	PtBiserial	5	0.520
20	Frey	2	2.381
21	McClain	2	0.186
22	Dunn	13	0.010
23	Hubert	0	0.000
24	SDindex	4	0.203
25	Dindex	0	0.000
26	SDbw	14	0.280

Conclusion: According to the majority rule, the best number of clusters is 2.

7 Model fit using NOAA RH and T for the 0.5-km radius dataset

To better estimate if NOAA meteorological data can replace PurpleAir meteorological data, we compared their DP since the water content and DP should be the same for the PurpleAir and the NOAA sites. Figure S5, which used all the hourly datapoints of our study, showed a Pearson correlation of 96 %. Except TX, which represented only 0.32 % of our dataset and exhibited a low correlation (13 %), all the NOAA sites resulted in a high correlation ranging from 80 to 97 % with PurpleAir sites.

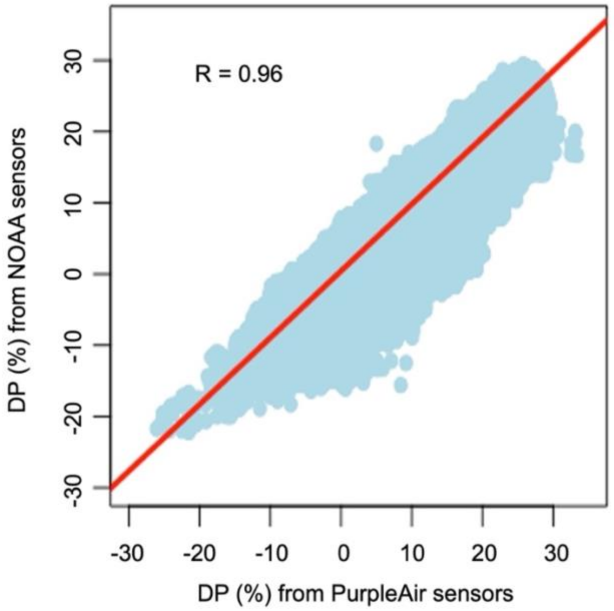

Figure S5: Correlation between DP from PurpleAir and NOAA

Table S10: MLR and SSC model development (model fit using hourly data) for the 0.5-km radius

Parameters		Model development				Sensitivity analysis			
Models		R^2 (%)	RMSE (μg m^{-3})	MAE (μg m^{-3})	R (%)	R^2 (%)	RMSE (μg m^{-3})	MAE (μg m^{-3})	R (%)
**MLR**	4.4968840+0.4184462 PA_i - 0.0353587 RH_i + 0.0779764 T_i	72	2.99	2.01	85	78	2.28	1.62	88
**SSC** (RH ≤ 50)	2.874778+0.461934 PA_i - 0.009394 RH_i + 0.077146 T_i	76	2.72	1.74	87	85	1.94	1.32	92
**SSC** (RH > 50)	5.6571930+0.4101217 PA_i - 0.0472842 RH_i + 0.0724931 T_i	72	3.03	2.05	85	77	2.31	1.64	88

8 Evaluation of the performance of the models by AQI category

Air Quality Index (AQI) is a communication tool used by EPA to inform public health. AQI for $PM_{2.5}$ is based on a 24-hour average with 6 categories of potential health impact: Good, Moderate, Unhealthy for sensitive groups, Unhealthy, Very Unhealthy, and Hazardous. They each correspond to a color code and a standard range of values.

Table S11 presents the distribution of all the evaluated data per AQI category. Table S12 shows the total percentage of correct AQI reported by each model with their under and over estimation. Models 4 and SSC reported the highest percentage of correct AQIs with a fairly even distribution of under- and overestimation shown by the SSC. Model Bj displayed a much higher underestimation than overestimation.

Table S11: Evaluation of the performance of the models by AQI category

Models	AQI definition	% of the data	Correct AQI (%)	Under-estimation (%)	Over-estimation (%)
SSC	Good (0.0 – 9.0 μg m^{-3})	60.66	86.56	0.00	13.44
Model 4			88.68	0.00	11.32
Model Bj			95.14	0.00	4.86
Raw PA			61.16	0.00	38.84
SSC	Moderate (9.1 – 35.4 μg m^{-3})	38.99	81.03	18.92	0.04
Model 4			77.95	22.05	0.00
Model Bj			66.77	32.96	0.27
Raw PA			91.99	7.74	7.88
SSC	Unhealthy for sensitive groups (35.5 – 55.4 μg m^{-3})	0.33	52.63	47.37	0.00
Model 4			52.63	47.37	0.00
Model Bj			78.95	15.79	5.26
Raw PA			0.00	0.00	100.00
SSC	Unhealthy	0.02	0.00	100.00	0.00
Model 4			0.00	100.00	0.00

Model Bj	(55.5 – 125.4 μg m^{-3})		0.00	100.00	0.00
Raw PA			100.00	0.00	0.00

Table S12: Summary table of the evaluation of the AQI per model for the daily dataset

Models	Correct AQI (%)	Under-estimation (%)	Over-estimation (%)
SSC	84.01	7.49	8.17
Model 4	84.10	8.70	6.87
Model Bj	83.78	12.81	3.07
Raw PA	72.68	2.99	26.94

9 Additional table

Table S13: List of the PurpleAir sensors and Federal Reference Method (FRM) or Federal Equivalence Method (FEM) used in the study with the estimated distance between stations

Site #	PA ID	PA latitude	PA longitude	AQS ID	FRM/FEM Type	Distance PA-AQS (km)	**Number PA-AQS Data points	NOAA ID	Distance PA-NOAA (km)
FL	25949	27.29050	-82.50697	121150013	Teledyne T640	0.028	13978	722115-12871	13.392
FL	16317	27.29050	-82.50830	121150013	Teledyne T640	0.123	21012	722115-12871	13.350
FL	101259	27.95523	-82.46953	120570113	Teledyne T640	0.011	2655	722110-12842	7.877
FL	149710	27.95523	-82.46956	120570113	Teledyne T640	0.011	3060	722110-12842	7.874
*GA	142428	33.77928	-84.39596	131210056	R & P Model 2025 PM-2.5 Sequential Air Sampler w/VSCC	0.500	-	722190-13874	17.434
*GA	148123	33.77932	-84.39611	131210056	R & P Model 2025 PM-2.5 Sequential Air Sampler w/VSCC	0.500	-	722190-13874	17.434
SC	35139	32.84358	-79.95844	450190020	Teledyne T640X	0.438	7264	722080-13880	10.972
NC	98623	35.24020	-80.78570	371190041	Met One BAM-1020	0.307	8495	723140-13881	18.780
NC	6008	36.11095	-80.22445	370670022	Teledyne T640X	0.005	19560	723193-93807	2.445
VA	178279	36.84141	-76.18123	518100008	Teledyne T640X	0.052	1109	723080-13737	7.038
TX	166421	29.82794	-95.28375	482010046	Met One BAM-1022	0.053	508	720594-00188	16.597
TN	176311	36.05266	-89.38216	470450004	Met One BAM-1022	0.033	2412	723347-03809	6.604
TN	93593	35.70589	-88.81981	471130010	Met One BAM-1022	0.066	2187	723346-03811	16.645
TN	51741	35.11688	-87.41976	470990003	Met One BAM-1022	0.004	18790	723235-13896	46.322
TN	51867	35.11688	-87.41972	470990003	Met One BAM-1022	0.001	20578	723235-13896	46.323
*TN	51737	35.11685	-87.41972	470990003	Met One BAM-1022	0.002	-	723235-13896	46.321
TN	93577	35.65182	-87.00883	471192007	Met One BAM-1022	0.086	7620	723249-00463	21.910
TN	93645	36.17619	-86.73885	470370023	Teledyne T640X	0.064	10893	723270-13897	9.235
TN	51921	36.17634	-86.73898	470370023	Teledyne T640X	0.058	6750	723270-13897	9.264
TN	51873	36.17625	-86.73911	470370023	Teledyne T640X	0.076	11779	723270-13897	9.262
TN	116559	36.17699	-86.74283	470370023	Teledyne T640X	0.474	998	723270-13897	9.589

* sensor removed after QA process

** valid data points used in our study after the QA process

Table S14: Tests of model coefficient precision

Number of significant figures	RMSE	MAE	R^2
Model 3			
8	2.318026	1.674111	0.7717575
4	2.318178	1.674819	0.7717097
3	2.320251	1.664604	0.7724541
Model 4			
8	2.236673	1.595438	0.7871297
4	2.237311	1.597079	0.7870842
3	2.244286	1.610342	0.7865547

References

U.S. EPA, https://document.airnow.gov/technical-assistance-document-for-the-reporting-of-daily-air-quailty.pdf, accessed on June 22, 2024
